# New focuses of clinical and translational medicine in 2020

**DOI:** 10.1002/ctm2.9

**Published:** 2020-03-31

**Authors:** Xin Cheng, Duojiao Wu, Yunfeng Cheng, Tiankui Qiao, Xiangdong Wang

**Affiliations:** ^1^ Centre for Tumor Diagnosis and Therapy Jinshan Hospital Fudan University Shanghai Medical College Shanghai China

**Keywords:** clinical trans‐omics, diagnosis, therapy

## Abstract

Clinical and translational medicine is an ongoing opportunity and challenge to transition science from bench to patient, from patient to bench, and from patient to policy for scientists and clinicians. Of the many objectives, the most important one is to improve the quality of life in patients, including living duration, dignity, emotional well‐being, psychiatric and physical health, as well as independency. Emphasis and redefining concepts has allowed clinical and translational medicine to experience exciting and productive developments. Further discoveries, innovations, validations, and developments in clinical and translational medicine are expected in 2020. The present editorial aims to present the top three most discussed topics with high expectations for translation from clinical investigations and trials into practice and application.

## 1 CLINICAL TRANS‐OMICS‐BASED DIAGNOSIS

Clinical trans‐omics is considered as an emerging technology and provides a multidimensional vision for identification and validation of new therapeutic targets and diagnostic biomarkers.^1^ The critical point of clinical trans‐omics is to identify the trans‐points or crossing‐points among different omics layer networks, especially the ones integrating clinical phenomes with molecular multi‐omics. The center of clinical trans‐omics is the position of clinical phenomics to define disease‐ and phenome‐specific biomarkers and targets, and benefit early diagnosis and therapeutic strategies.^2^ Additionally, clinical trans‐omics is strongly suggested to be able to identify new networks of molecule‐based function, the critical point of the disease progression, and new evidence for precision medicine‐required therapies. The development of drug resistance, for example, chemotherapy, target drugs, or immunotherapy, is a secondary threat and varies among individuals during treatment. Clinical trans‐omics is expected to illuminate three‐ or four‐dimensional mechanisms by which drug resistance occurs or can be prevented.

## 2 HUMAN ORGANOIDS‐BASED DRUG SCREENING

Functioning human or disease‐specific organoids are rapidly developing and improving to demonstrate structural and functional properties of human organs or dynamic and pathophysiological characteristics of human diseases,^3^, ^4^ for example, cancer modeling. Human organoids can model human organ evolution and development and various human pathologies in well‐controlled conditions, especially disease‐specific organoids. Human organoids are mainly applied as a model for human‐specific organs and cancer evolution or drug response evaluation, as a living biobank for heterogeneity detection or drug efficacy screening, and as a mini‐bioreactor for mechanism‐based investigations of cell‐cell communication, tumor immune microenvironment, and drug discovery. Furthermore, organoids provide the potential to dynamically monitor the interaction between the disease‐specific circulating cells and the corresponding/matched pathological organoids. For example, tumor organoids were co‐cultured with peripheral blood lymphocytes from peripheral blood of patients with cancer to develop tumor‐reactive or tumor‐infiltrated T cells and assess the sensitivity of patient‐specific tumor cells to T‐cell‐mediated toxicity.^1^ Human organoids can be applied to clinical practice to predict the occurrence of drug efficacy and resistance, develop personalized and precise therapy, and engineer the compromised tissues/organs in the individual patient. The application of human organoids further extended by integrating them with other advanced technologies, for example, genome editing to establish the model of genetic disorders. There is an urgent need to standardize the process of human‐specific or disease‐specific organoids, monitor the quality and quantity of organoid cells, and confirm the repeatability of the information generated from organoids.

## 3 HUMAN GENE EDITING–BASED THERAPY

Gene editing, with rapid development and maturation of genome editing technologies, is strongly suggested as a precise and efficient alternative to clinical therapies for human diseases. Gene editing is one of the most wanted therapies in clinical and translational medicine, although there are still many challenges and obstacles to be faced and overcome. The number, precision, and consequence of off‐targets after gene editing need to be furthermore clarified and defined before any clinical practice. A recent study suggests that prime editing is a versatile and precise genome editing method in human cells to achieve the definite modification in the specified DNA site.^5^ The prime editing guide RNA can precisely define the target site and modify the desired encodes using a catalytically impaired Cas9 endonuclease fused to an engineered reverse transcriptase. This is a revolutionary improvement to maintain high efficiency and fewer byproducts as well as a much lower occurrence of off‐target editing. The most important issue in clinical application of gene editing is to specify the clinical indications and patient populations, ensure the quality and stability of the methodology, and establish the international ethics and regulation. A strong call for “a global moratorium on all clinical uses of human germline editing” was recently made by the global opinion leaders in the field of gene editing, especially with regard to editing heritable DNA (in sperms, eggs, or embryos).^6^ This is the vital issue to be clarified before any of the clinical applications and trials occur, and needs to be urgently agreed upon within and among nations and formalized with laws and regulations. Gene editing has been considered as an alternative therapy for patients with mono‐gene disorders or cancers, although more obstacles need to be overcome.

Besides three major progresses or trends discussed above, artificial intelligence (AI) is the development of computer systems that has been recently applied in clinical research and applications.^7^, ^8^ For example, AI is explored in gastroenterology for endoscopic analysis of lesions. Inflammatory lesions or gastrointestinal bleeding analysis during endoscopy could be used for cancer detection and to differentiate patients with cancer from those with chronic inflammation.^9^ Advances in technology in AI, especially deep‐learning algorithms and the graphics‐processing units (GPUs), contribute to a recent and rapidly increasing interest in medical AI applications, including clinical diagnostics, and processing large and complex genomic datasets. It is powerful for calling variant, annotating genome data, and linking the data between phenotype and genotype. Therefore, we expect the applications of AI in enhancing the understanding of diseases and the deep learning frameworks for further investigation.

When we retrospectively reviewed the journal of Clinical and Translational Medicine (CTM) published during 2016‐2019, we found that *CTM*’s main focuses were on cancer biomarkers and therapeutic strategies in about 27% of publications; molecular regulations at DNA, RNA, and nuclear levels in 22%; intra‐ and intercellular and organelle functions in 23%; and metabolism‐related dysfunction in 14%, of which 34% were original articles and 40% were review articles. Approximately 820 scientists and clinicians contributed to the development of CMT as authors and the majority (43%) came from the United States. The continental breakdown of those articles is 50% from North America, 25% from Europe, and 20% from Asia. Total download and citation times are 385 104 and 1585 according to the preliminary calculation. On the basis of those successes, CTM has started to have its own strategy for further development with its own special focuses.

In conclusion, clinical and translational medicine calls special attention from scientists and clinicians in 2020 for a multitude of topics, for example, clinical trans‐omics‐based diagnosis, human organoids–based drug screening, and human gene editing–based therapy. We expect and look forward to articles on advanced improvements of related methodologies, development strategies of translational processes into clinical practice, clinical trials of disease‐specific biomarkers and therapies, and clinical evaluations of biomarker and drug discoveries.
